# Supplementary material for: Advanced Characterization of Imiquimod-Induced Psoriasis-Like Mouse Model
Source: Pharmaceutics. 2020 Aug 20;12(9):789. doi: 10.3390/pharmaceutics12090789 (PMC7558091; doi:10.3390/pharmaceutics12090789)
Supplement: Supplementary file 1 [file pharmaceutics-12-00789-s001.pdf]

pharmaceutics

MDPI

Supplementary Materials: Advanced Characterization of Imiquimod-Induced Psoriasis-Like Mouse Model

Mehwish Jabeen, Anne-Sophie Boisgard, Alix Danoy, Naima El Kholti, Jean-Paul Salvi, Roselyne Boulieu, Bérengère Fromy, Bernard Verrier and Myriam Lamrayah

Skin perfusion using Laser Speckle Contrast Imaging (LSCI)

Methods: In order to measure skin perfusion at the psoriatic sites (back and right ear), as well as for negative control (left ear), the non-invasive Laser Speckle Contrast Imaging (PeriCam PSI System®, Perimed, France) was used as previously described in human and animal studies [1,2]. Two images per second were taken and the average of skin perfusion was calculated using the manufacturer's software (PimSoft5.1®; Perimed). Results are expressed in arbitrary units (a.u.).

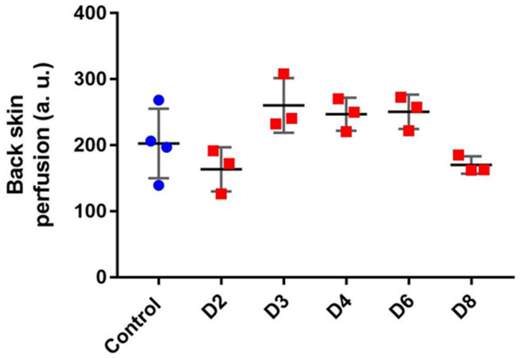

Figure S1. Measurement of back skin perfusion for 8 days with daily IMQ application. Evaluations were statistically analyzed using a one-way ANOVA followed by Dunnett multiple comparison test, all comparisons were not significantly different. Values are presented as mean ± SD (n=3, except for control where n=4).

No statistical differences are observed for the back skin perfusion following IMQ application, probably due to the low number of mice in each group. The inter-individual variability is undoubted and masks the evolution of the parameter attributable to the phenotype. There is, however, a tendency to an increased skin perfusion from day 3 to day 6, which is in accordance with the redness evolution measured through PASI score and the appearance of dermal hypervascularity or angiogenesis markers.

Maintenance of the psoriatic phenotype in the IMQ-induced psoriasis model during 8 days of IMQ application and 15 days of observation

Methods: The experimental procedure was the same as already described in the main manuscript (see section 2.2), except that the study was not stopped at day 8. The mice were kept alive for seven more days after discontinuation of IMQ application, to examine the maintenance of the psoriatic phenotype in this model.

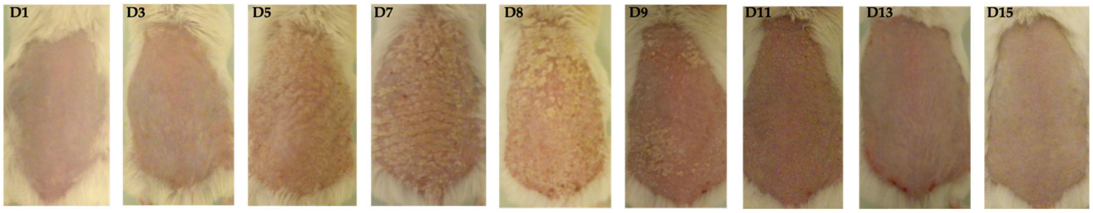

(**A**)

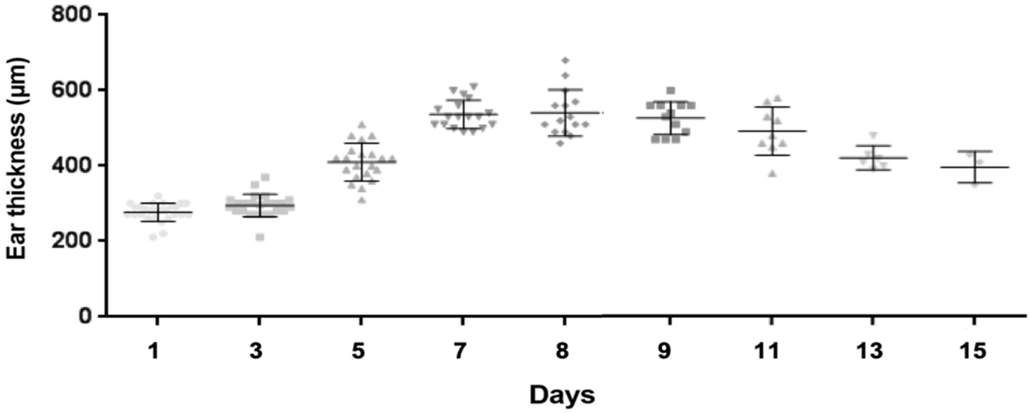

(**B**)

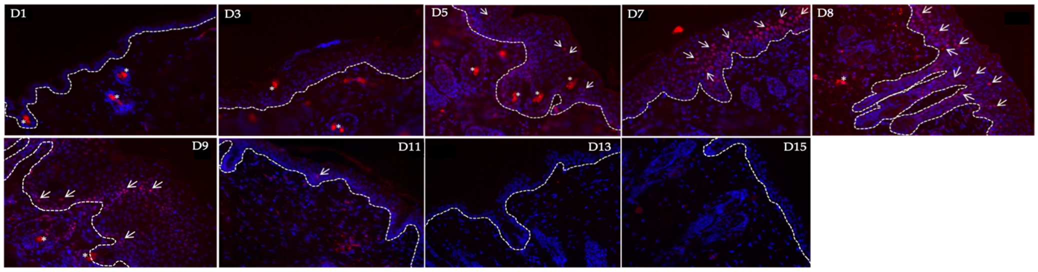

(**C**)

Figure S2. Loss of psoriasis-like phenotype after day 8. **A**) Evaluation of dorsal skin during 15 days, with IMQ cream application from day 1 to day 8; **B**) Evaluation of right ear thickness during 15 days with daily IMQ application from day 1 to day 8. Values are presented as mean ± SD (n=27 for day 1 and decreased subsequently to n=3 for day 15); **C**) pSTAT3 staining evaluation on dorsal skin during 15 days, with IMQ cream application from day 1 to day 15. The dotted white line represents the dermo-epidermal junction with dermis below. pSTAT3 protein appears in red (indicated by arrows) and cell nuclei in blue (asterisks represent the unspecific red staining).

IMQ-induced psoriasis mouse model to evaluate anti-psoriatic treatment

Methods: The experimental procedure was the same as already described in the main manuscript (see section 2.2). The animals were divided into four groups (n=4). Briefly, the mice were shaved and depilated on their back using a Wahl Brav Mini Clipper razor and commercially available depilatory cream (Veet®). The experimental groups were treated daily with application of 62.5 mg of commercially available 5% IMQ cream (Aldara, MEDA Pharmaceuticals) at the depilated back, during eight days. In parallel, twelve hours after IMQ application, the groups IMQ+ tofacitinib and IMQ + dermoval received respectively 125 mg of a cream containing 0.1% tofacitinib or 125 mg of a cream containing 0.05% of clobetasol propionate (Dermoval, GSK). Dermoval was used here as positive control of psoriasis topical treatment [3]. The control group was treated similarly with Lanette cream containing cetyl stearyl alcohol (BASF).

The tofacitinib cream was prepared by dissolving 15 mg of tofacitinib citrate (Sigma) in 10 mL of pure water and 5 mL of excipient Versatile (Fagron), then the mixture was homogenized.

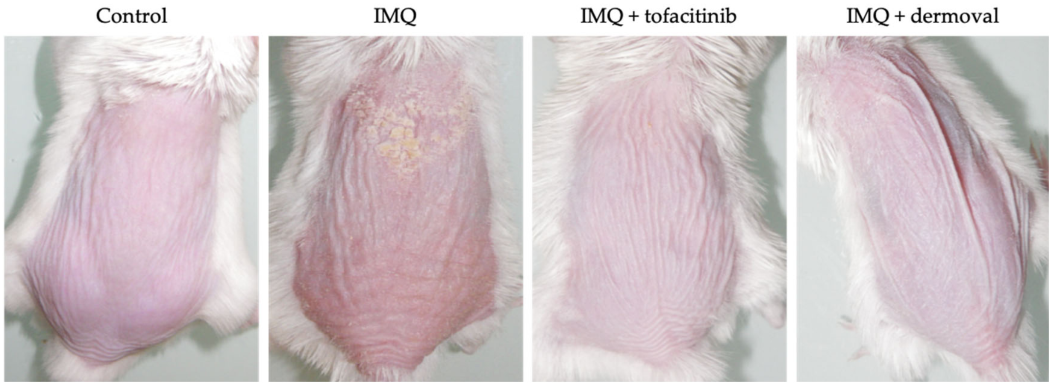

(A)

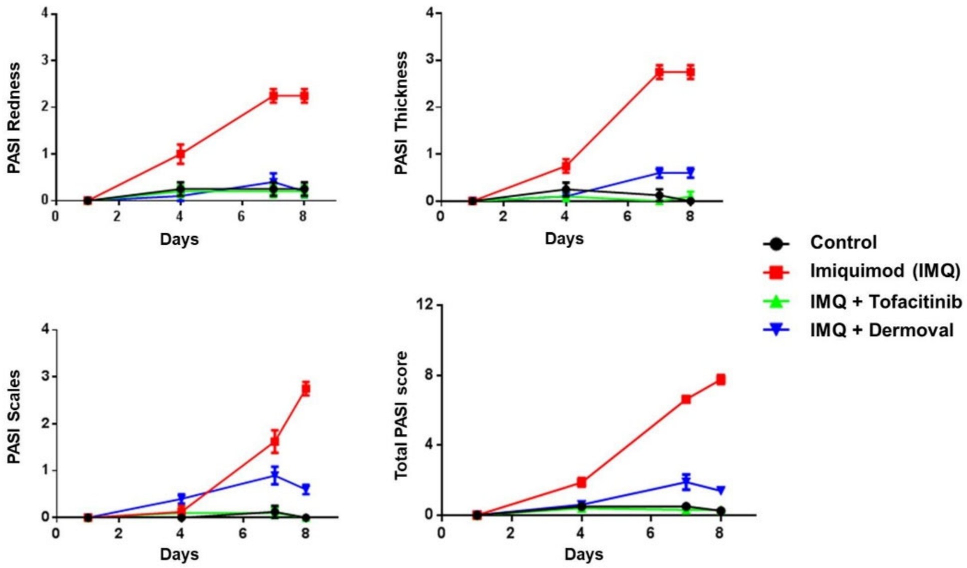

.

(B)

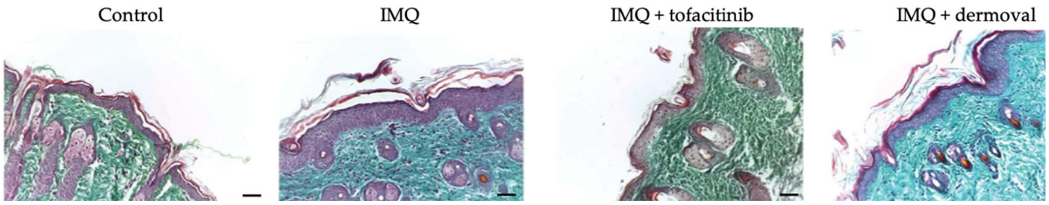

.

(C)

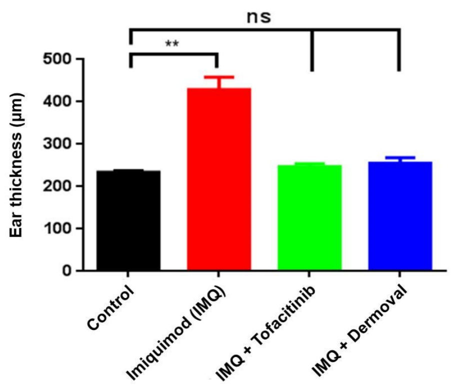

(D)

Figure S3. Application of the IMQ-induced psoriasis mouse model previously characterized as a tool to assess anti-psoriatic treatment efficacy. **A**) Evaluation of dorsal skin at day 9 for the four groups; **B**) Evaluation of PASI score in the course of the evaluation of psoriatic treatment on IMQ-induced psoriasis model. Observed criteria are skin redness, thickness, the presence of scales and total PASI score; **C**) Histological examination by Masson's trichrome staining of back skin samples (scale bar = 100 µm for each image); **D**) Measurement of ear thickness at day 9 on control and IMQ-induced mouse model with or without treatments. Results were statistically analyzed using one-way ANOVA followed by Dunnett's multiple comparison test. Values are presented as mean ± SD. ** : $p<0.001$.

References

1. Fromy, B.; Lingueglia, E.; Sigaudo-Roussel, D.; Saumet, J.L.; Lazdunski, M. Asic3 is a neuronal mechanosensor for pressure-induced vasodilation that protects against pressure ulcers. *Nat. Med.* **2012**, *18*, 1205–1207, doi:10.1038/nm.2844.
2. Tew, G.A.; Klonizakis, M.; Crank, H.; Briers, J.D.; Hodges, G.J. Comparison of laser speckle contrast imaging with laser Doppler for assessing microvascular function. *Microvasc. Res.* **2011**, *82*, 326–332, doi:10.1016/j.mvr.2011.07.007.
3. Sun, J.; Dou, W.; Zhao, Y.; Hu, J. A comparison of the effects of topical treatment of calcipotriol, camptothecin, clobetasol and tazarotene on an imiquimod-induced psoriasis-like mouse model. *Immunopharmacol Immunotoxicol* **2014**, *36*, 17–24, doi:10.3109/08923973.2013.862542.
